# HIV-1 gp120 and morphine induced oxidative stress: role in cell cycle regulation

**DOI:** 10.3389/fmicb.2015.00614

**Published:** 2015-06-23

**Authors:** Thangavel Samikkannu, Deepa Ranjith, Kurapati V. K. Rao, Venkata S. R. Atluri, Emely Pimentel, Nazira El-Hage, Madhavan P. N. Nair

**Affiliations:** Department of Immunology, Institute of NeuroImmune Pharmacology, College of Medicine, Florida International University, Miami, FL, USA

**Keywords:** HIV-1 gp120, morphine, oxidative stress, cell cycle and microglia

## Abstract

HIV infection and illicit drugs are known to induce oxidative stress and linked with severity of viral replication, disease progression, impaired cell cycle regulation and neurodegeneration. Studies have shown that morphine accelerates HIV infection and disease progression mediated by Reactive oxygen species (ROS). Oxidative stress impact redox balance and ROS production affect cell cycle regulation. However, the role of morphine in HIV associated acceleration of oxidative stress and its link to cell cycle regulation and neurodegeneration has not been elucidated. The aim of present study is to elucidate the mechanism of oxidative stress induced glutathione synthases (GSS), super oxide dismutase (SOD), and glutathione peroxidase (GPx) impact cell cycle regulated protein cyclin-dependent kinase 1, cell division cycle 2 (CDK-1/CDC-2), cyclin B, and cell division cycle 25C (CDC-25C) influencing neuronal dysfunction by morphine co-morbidity with HIV-1 gp120. It was observed that redox imbalance inhibited the GSS, GPx and increased SOD which, subsequently inhibited CDK-1/CDC-2 whereas cyclin B and CDC-25C significantly up regulated in HIV-1 gp120 with morphine compared to either HIV-1 gp120 or morphine treated alone in human microglial cell line. These results suggest that HIV positive morphine users have increased levels of oxidative stress and effect of cell cycle machinery, which may cause the HIV infection and disease progression.

## Introduction

HIV infection is a major cause of human death and globally it was estimated that 35 million people are living with HIV (UNAIDS/WHO, 2014). HIV comorbidity with illicit drugs remains a global health problem. Illicit drugs of abuse including opiate is a significant risk factor for HIV infection and AIDS disease progression (Hu et al., 2012; Masvekar et al., 2014; El-Hage et al., 2015). Opiate causes deterioration of neuronal functions in a significant proportion of users resulting in the development of neuronal impairments and HIV associated neurocognitive dysfunction (HAND). Previous studies demonstrate that heroin is deacetylated to morphine, which can affect the central nervous system (CNS), and is very often associated with HIV infection and activated macrophages/microglia, are the major reservoir of viral replication and disease progression (Navia et al., 1986; Kure et al., 1990; Brzezinski et al., 1997). HIV-1 infection is known to cause CNS dysfunction and primarily affects microglial cells and subsequently impact astrocytes and neurons. Clinical observations suggest that selective regions of brain such as the striatum and the hippocampus highly express opioid receptors and have been associated with increased viral titers in HIV-infected patients (Mansour et al., 1987; Mansour and Watson, 1993).

The HIV-1 envelop protein, glycoprotein 120 (gp120) is required for viral entry and it facilitates the viral replication and disease progression mediated by oxidative stress, which subsequently affects the CNS (Brenneman et al., 1994; Galicia et al., 2002). Previous studies have shown that HIV-1 gp120 associated with increased secretion of inflammatory cytokines and chemokines (Holguin et al., 2004), glutamate, arachidonic acid and its metabolites prostaglandin E2 (PGE_2_) and 5-lipoxygenase (5-LOX; Samikkannu et al., 2011), nitric oxide (NO) and reactive oxygen species (ROS; Ronaldson and Bendayan, 2008; Lisi et al., 2014). In microglia, gp120 induces oxidative stress, ROS, TNF-*α*, and MCP-1 production and subsequently leading to neuronal cell death (Guo et al., 2013). Also, morphine was found to induce oxidative stress in *in vitro* as well as *in vivo* (Kovacic and Cooksy, 2005; Ma et al., 2015). Studies have also shown that morphine impaired cellular function mediated by oxidative stress which affects the CNS (Moron et al., 2009; Beltran-Campos et al., 2015).

Studies have documented that increased level of oxidative stress and ROS is associated with HIV-associated neurocognitive impairment (Shah et al., 2013). Free radicals H_2_O_2_ and O_2_^–^, induced by oxidative stress are known to generate cellular damage in multiple diseases, including HIV/AIDS (Kashou and Agarwal, 2011). Glutathione (GSH) is a tri peptide and known to be important cell protectant in intracellular anti-oxidant defense mechanisms, and its reduced levels have been correlated with impaired neuronal function (Aoyama et al., 2008). However, oxidative stress and cell cycle reentry seems counterintuitive. Previous report demonstrated that oxidative stress on the cell cycle reveals that increased ROS-induced DNA damage is correlated with cell cycle arrest (Pyo et al., 2013). However, whether ROS-induced stress response undergoes growth arrest or apoptosis may depend in part on where the cell resides in the cell cycle when insulted. Extensive studies have consistently demonstrated that morphine consumption and HIV infection further stimulate the sensitivity and neuronal dysfunction severity that causes HAND (Grigoryan et al., 2010; Masvekar et al., 2014). HIV infected morphine users have synergistically potentiated viral replication and disease progression as compared with HIV positive subjects (Roy et al., 2011; Dutta and Roy, 2012; Masvekar et al., 2014; El-Hage et al., 2015). Despite mounting evidence suggesting morphine use may aggravate HIV infection, mechanistic studies determining the mutual role of morphine and HIV infection on cell cycle regulation and their role in neurodegeneration are yet to be determined. Therefore, we have investigated the mechanism of HIV-1gp120 induced oxidative stress altered cell cycle regulation and morphine co-morbidity accelerated effects in microglial cells.

In this study, we have investigated the effect of HIV-1 gp120 in combination with morphine induced oxidative stress that affect the redox expression in GSS, SOD, GPx, and cell cycle arrest in G0 phase leads CDK-1/CDC-/2, cyclin B, and CDC-25C associated neuropathogenesis. We showed that morphine with HIV-1 gp120 exacerbates the oxidative stress and cell cycle arrest in microglial mediated CNS dysfunction.

## Materials and Methods

### Cell Culture and Reagents

Cell culture reagents were purchased from Sciencell (Carlsbad, CA, USA). The glutathione synthetase (GSS), super oxide dismutase (SOD), glutathione peroxide (GPx), and CDC25C antibodies were purchased from Santa Cruz Biotechnology (Santa Cruz, CA, USA). The mouse anti-cyclin B and CDK-1/CDC-2 antibodies were purchased from BD Transduction Laboratories (San Jose, CA, USA). The goat anti-rabbit IgG and goat anti-mouse IgG antibodies were purchased from Santa Cruz Biotechnology. The propidium iodide was purchased from PD bioscience (San Jose, CA, USA). Electrophoresis reagents were purchased from Bio-Rad (Richmond, CA, USA), and nitrocellulose membranes were purchased from Amersham Scientific, Piscataway, NJ, USA. All other reagents were purchased from Sigma–Aldrich (St. Louis, MO, USA).

### HIV-1 gp120 Recombinant Proteins

The HIV-1 gp120 (Bal) protein was obtained from the NIH AIDS Research and Reference Reagent Program. The recombinant gp120 proteins were tested >95% purity.

### Microglial Cell Line (CHEM-5) Cultures

In this study, we used immortalized microglial CHEM-5 cells. Microglial cells were maintained in Dulbecco’s Modified Eagle Medium supplemented with fetal bovine serum to a final concentration of 10% (catalog # 30- 2020) and 1% antibiotic/antimycotic solution (Sigma-Aldrich, St. Louis, MO, USA).

### RNA Extraction and Real time Quantitative PCR (qRT-PCR)

Total RNA from microglia was extracted using the Qiagen RNAeasy mini kit (Invitrogen Life Technologies, Carlsbad, CA, USA) by following the manufacturer’s instructions. The total RNA (3 μg) was used for the synthesis of cDNA. The amplification of cDNA was performed using specific primers for GSS (Assay ID Hs00609286_m1), GPx (Hs01591589_m1), and SOD (Hs00166575_m1); and β-actin (Hs99999903_m1) (Applied Biosystems, Foster City, CA, USA) was used as a housekeeping gene for quantifying real-time PCR. Relative abundance of each mRNA species was assessed using brilliant Q-PCR master mix from Stratagene using Mx3000P instrument that detects and plots the increase in fluorescence versus PCR cycle number to produce a continuous measure of PCR amplification. Relative mRNA species expression was quantitated and the mean fold change in expression of the target gene was calculated using the comparative CT method (Transcript Accumulation Index, TAI = 2^_ΔΔCT^). All data were controlled for quantity of RNA input by performing measurements on an endogenous reference gene, β-actin. In addition, results on RNA from treated samples were normalized to results obtained on RNA from the control, untreated sample.

### Western Blot Analysis

To determine the GSS, SOD, GPx, CDK-1/CDC-2, cyclin B, and CDC25C protein alterations in microglial control, morphine, HIV-1 gp120 and morphine with HIV-1 gp120 treated cells, equal amount of total cellular protein were resolved on a 4–15% gradient polyacrylamide gel electrophoresis, transferred to a nitrocellulose membrane and incubated with their respective primary and secondary antibodies. Immunoreactive bands were visualized using a chemiluminescence western blotting system according to the manufacturers’ instructions (Amersham).

#### Analysis of HIV-1 gp120 and Morphine Impact on Cell Cycle Regulation

Morphine and HIV-1 gp120 induced DNA damage and cell cycle arrest were analyzed by flow cytometry in FACS caliber (BD Bioscience, San Jose, CA). Briefly, Microglial (5 × 10^5^) were separately treated with morphine, HIV-1 gp120, and HIV-1 gp120 with morphine. Cells were harvested and washed twice with PBS. The cells were fixed in 0.5% paraformaldehyde and stained with PI solution after 24 h. The ModFit LT software program (Verity Software House) was used to determine the distribution of cells in subG0/G0 phase of the cell cycle.

#### Data Analysis

Statistical analysis was performed using GraphPad Prism- version 5. Differences between morphine, HIV-1 gp120 and morphine with HIV-1 gp120 treated and control were calculated using two-way ANOVA method. Values were expressed as mean ± standard error and a significance level of *p* < 0.05 was used.

## Results

### HIV-1 gp120 and Morphine Induced Oxidative Stress; and Redox Gene and Protein Expression in Microglial Cells

**FIGURE 1:**
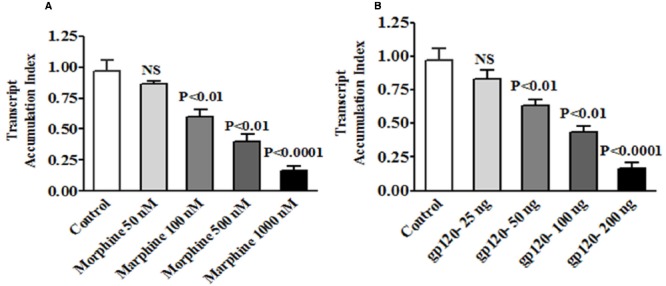
**The kinetic studies of Morphine and HIV-1 gp120 protein impact redox dysfunction in GSS gene expression.** Microglia (1 × 10^6^ cells/ml) were treated with **(A)** morphine (0–1000 nM) and **(B)** HIV-1 gp120 (0–200 ng) for 24 h for dose response studies. RNA was extracted, reverse transcribed, and subjected to quantitative real-time PCR using specific primers for GSS and the housekeeping gene β-actin. Data are expressed as the mean ± SE of the TAI values from three independent experiments.

**FIGURE 2:**
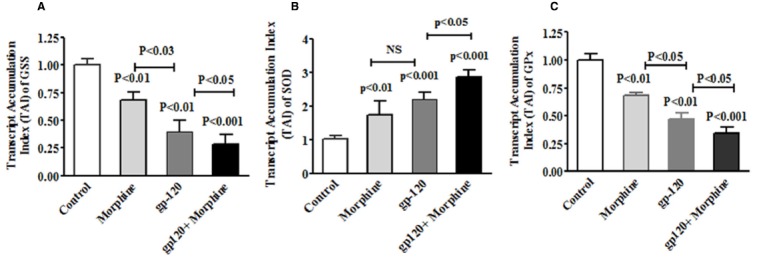
**HIV-1 gp120 and morphine effect on redox genes GSS, SOD and GPx expression.** Microglia (1 × 10^6^ cells/ml) were treated with HIV-1 gp120 (100 ng), morphine (0.5 μM) and HIV-1 gp120 with morphine. Controls were maintained by drug free medium. The end of the incubation, RNA was extracted and reverse transcribed followed by quantitative real time PCR for GSS **(A)**, SOD **(B)**, GPx **(C)**, and housekeeping β-actin specific primers. Data are expressed as mean ± SE of TAI values of three independent experiments.

It is known that HIV infection; HIV-1 gp120 protein and morphine induced ROS production lead to cell death (Ronaldson and Bendayan, 2008; Ma et al., 2015). However, no information is available on HIV-1 gp120 and morphine induced ROS production and redox altered cell cycle arrest and neurodegeneration. Therefore, we determined whether HIV-1 gp120 induce redox expression, cell cycle regulation and neurodegeneration are accelerated by morphine in microglia. Observed results demonstrate that HIV-1 gp120 and morphine significantly increased oxidative stress and subsequently inhibited the redox expression in microglia. The data presented in Figures 1A,B show the dose response in morphine- (0–1000 nm/ml) and gp120—(0–200 ng/ml) treatment for 24 h and its effects on GSS gene expression in microglia, as assessed using quantitative real-time PCR. Microglia treated with morphine significantly down regulated GSS gene expression at 100 nm (*p* < 0.01), 500 nm (*p* < 0.001) and 1000 nm (*p* < 0.0001) compared to control. In HIV-1 gp120 effect on 50 ng (*p* < 0.01), 100 ng (*p* < 0.001) and 200 ng (p < 0.0001) compared to control was observed respectively. Figure 2 shows the effects of HIV-1 gp120, morphine and HIV-1 gp120 with morphine impact on redox gene GSS (A), SOD (B), and GPx (C) expression. These studies showed that HIV-1 gp120 and morphine treatment inhibit the levels of GSS and GPx whereas SOD significantly increased, and the combination of HIV-1 gp120 with morphine accelerates these effects compared with control. The present finding suggested that HIV-1 gp120 with morphine effects are accelerated as compared to either HIV-1 gp120 or morphine alone (Figures 2A–C).

**FIGURE 3:**
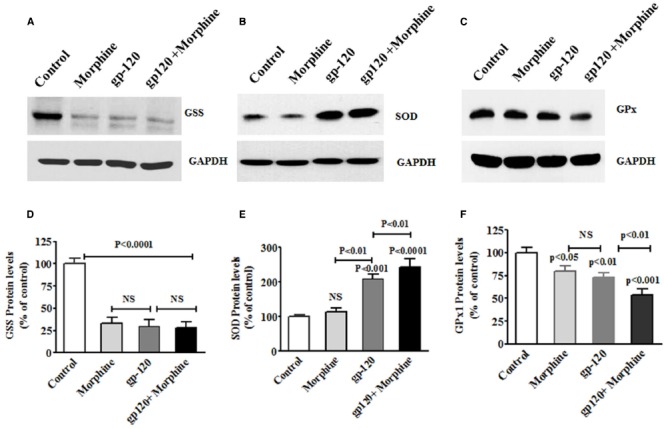
**HIV-1 gp120 with morphine effect on redox expression.** Microglia (1 × 10^6^ cells/ ml) were treated with HIV-1 gp120 (100 ng), morphine (0.5 μM) and combination of HIV-1 gp120 with morphine. Controls were maintained by drug free medium. The end of the incubation, equal amount of protein lysate were resolved by 4–15% SDS-PAGE and protein expression were analyzed by Western blot showing GSS **(A)**, SOD **(B)**, and GPx **(C)**. **(D–F)** represented % densitometric values of GSS, SOD, and GPx protein levels (% control). Data are expressed as mean ± SE of three independent experiments.

Furthermore, we also examined whether HIV-1 gp120 induced oxidative and redox protein regulation is accelerated by morphine. Figure 3 shows the effects of HIV-1 gp120, morphine and HIV-1 gp120 with morphine effects on redox protein GSS (A), SOD (B), and GPx (C). These observed results confirm that HIV-1 gp120 induced redox changes were accelerated by morphine. Taken together, these finding suggested that oxidative stress induced redox changes in HIV infection along with morphine leads to neurodegeneration.

### HIV-1 gp120 and Morphine Effects Cell Cycle Protein in Microglial cells

**FIGURE 4:**
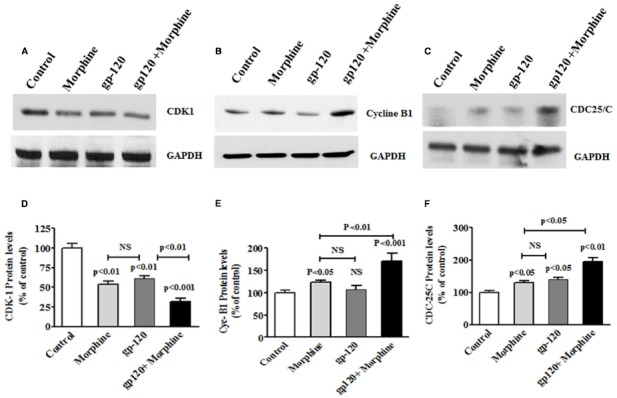
**HIV-1 gp120 with morphine effect on cell cycle proteins.** Microglia (1 × 10^6^ cells/ml) were treated with HIV-1 gp120 (100 ng), morphine (0.5 μM) and combination of HIV-1 gp120 with morphine. Controls were maintained by drug free medium. The end of the incubation, equal amount of protein lysate were resolved by 4–15% SDS-PAGE and protein expression were analyzed by Western blot showing cycline B1 **(A)**, CDK1 **(B)**, and CDC25C **(C)**. **(D–F)** represented % densitometric values of Cyc-B1, CDK1, and CDC25C protein levels (% control). Data are expressed as mean ± SE of three independent experiments.

Oxidative stress is known to induce DNA damage and cell cycle arrest. Therefore, we wanted to test whether exposure to HIV-1 gp120 protein affect cell cycle protein and neurodegeneration. In the present study, HIV-1 gp120 with morphine treated microglial cells showed a significant decrease in CDK-1/CDC-2 whereas cyclin B and CDC25C increased protein expression at 24 h, compared with either HIV-1 gp120 or morphine alone. Figure 4 shows HIV-1 gp120 with morphine effect on CDK1/CDC-2 (A), cyclin B (B), and CDC-25C (C) protein expression as compared to the control. These observations confirmed that morphine accelerates HIV-1 gp120 induced cell cycle effect.

### HIV-1 gp120 and Morphine Accelerates Cell Cycle Arrest

**FIGURE 5:**
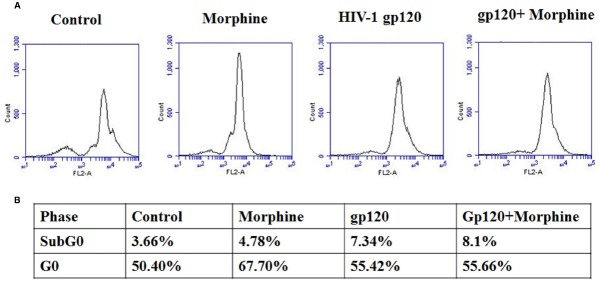
**HIV-1 gp120 with morphine effect on cell cycle progression.** Microglia (1 × 10^6^ cells/ml) were treated with HIV-1 gp120 (100 ng), morphine (0.5 μM) and combination of HIV-1 gp120 with morphine and its effect on cell cycle transitions were analyzed by flow cytometry **(A)** and cell cycle phase are expressed as % **(B)**.

HIV infection affects cell cycle regulation and ultimately leads to CNS dysfunctions (Amini et al., 2004; Zhao and Elder, 2005; Liu et al., 2007; Wang et al., 2010). Microglia is one of the major reservoirs of HIV infection and target of illicit drugs in the CNS. Therefore, we examined the effect of HIV-1 gp120 with morphine on cell cycle regulation in microglia. Figure 4 showed that HIV-1 gp120 and morphine treated microglia significantly inhibited cells at subG0 and G0 phase as compared with either HIV-1 gp120 or morphine alone (Figure 5). This study suggested that oxidative stress induced redox inhibition subsequently lead cell cycle arrest and this effect might be accelerated by morphine mediated neurodegeneration.

## Discussion

Studies have shown that oxidative stress plays a wide role in cellular function including immune and neuronal dysfunction, behavioral impairments in HIV-infection and substance abuse (Grigoryan et al., 2010; Masvekar et al., 2014); El-Hage et al., 2015). The oxidative stress induced ROS production is associated with HAD or HAND. Glutathione is an important player in the regulation of redox functions in cellular homeostasis (Schafer and Buettner, 2001). Studies have shown that HIV infection induced oxidative stress and ROS production subsequently affect redox proteins such as GSS, SOD and GPx in HIV infected patients and morphine users (Herzenberg et al., 1997; Koutsilieri et al., 1997; Bhaskar et al., 2015). However, overstimulation of ROS leads to inhibition in redox status GSH/GSSG (Koutsilieri et al., 1997), and subsequently impact the cell cycle machinery in cyclin B, CDK-1/CDC-2 and CDC-25C, which may play a vital role in neuronal dysfunction and disease progression in HAD patients (Klein and Ackerman, 2003; Morris et al., 2012). The CNS microglia cells are the major reservoirs of HIV infection and disease progression; they are modulated by cell cycle arrest induced by CDK-1/CDC-2, cyclin B and CDC-25C that lead to neurodegeneration (Cernak et al., 2005; Wang et al., 2008). However, there are no reports on the effects of HIV-1 gp120 and morphine induced redox imbalance that initiate cell cycle arrest and cellular mechanisms of neurodegeneration. The observed results provide new insights into the functional role of redox expression, which subsequently affects cell cycle machinery in HIV-1 gp120 with morphine.

In the present study, we have demonstrated for the first time that morphine, HIV-1 gp120 and HIV-1 gp120 with morphine showed increased oxidative stress and inhibit redox levels of GSS and GPx and, subsequently increased SOD mRNA and protein expression (Figures 1–3), associated with increased ROS production as compared to the control. It is known that redox dysfunction and GSH/GSSG ratio are the major players in maintaining cellular homeostasis. These studies suggest that morphine with HIV–1 gp120 may have an enhanced role of on oxidative stress as compared to the control. This is consistent with earlier reports of gp120 and morphine induced oxidative stress in microglia and macrophages, respectively where activation of the redox pathway has been observed (Bhat et al., 2004; Ronaldson and Bendayan, 2008; Lisi et al., 2014). Also, studies have shown that oxidative stress and redox impairments are associated with cell cycle arrest and DNA damage in the cerebral cortex brain regions (Klein and Ackerman, 2003). These studies further confirm that the cell cycle process in sub G0 and G0 stage are affected by morphine with HIV-1 gp120 and leading to neurodegeneration.

Moreover, our results show that in morphine, HIV-1 gp120, and morphine with HIV-1 gp120 induction of DNA damage and cell cycle arrest (Figures 4 and 5) is associated with inhibition of CDK-1/CDC-2 and subsequently increased cyclin B1 and CDC-25C. The main observation in this report is that HIV-1 gp120, and morphine with HIV-1 gp120 have higher impact on cell cycle arrest in the G0 phase due to ROS production and subsequently reduced the level of redox expression and CDK-1 inhibition. However, the HIV positive morphine users have higher ROS levels and DNA damage. This suggests that there is an interactive role between morphine and HIV that synergistically potentiates and increases co-morbidity when compared to either morphine use or HIV infection. These results confirm the previous report of HIV-1 gp120 induced cell cycle arrest in CD4^+^ T Cells (Kolesnitchenko et al., 1995).

Previous studies demonstrated that HIV-Vpr and Nef proteins affect cell cycle regulation which may impact cell cycle process (Yang et al., 2002; Wen et al., 2007; Li et al., 2010). In the present study, HIV-1 gp120 with morphine showed inhibition of CDK-1/CDC-2 and subsequent activation of cyclin B1 and CDC-25C protein as compared to the control. Also, cell cycle arrest in the G0 phase inhibited CDK-1/CDC-2 phosphorylation in microglia could lead to an enhanced DNA damages, which alters neuronal function and resulting in neurodegeneration. Furthermore, it is known that Gi/Gq-induced synergistic activation of ERK and subsequently affect between Ca^2+^ and Src family tyrosine kinases (Chan et al., 2005). However, Morphine and HIV-1 gp120 also known to activate ERK and downstream effect on CREB transcription mediated by Ca^2+^. Also, HIV-1 gp120 induced cell cycle arrest and these effects are accelerated by morphine in additive manner and this effect at Gi/Gq phase. These results suggest that viral replication in HIV infected microglial play a wide role in neuronal dysfunction, once HIV-1 gp120 or morphine effects altered DNA damages and subsequently increased CDC-25C in cell cycle arrest mediated by cyclin B.

Overall, the data provides evidence of interaction of morphine and HIV-1 gp120 leading to increase in oxidative stress and redox inhibition which is associated with increased levels of DNA damage and subsequently affecting cell cycle process. Based on these results, redox imbalance potentiates the HIV viral replication and disease progression by impairing the neuronal function, which may lead to neurodegeneration. The present study supports some of earlier reports in the dysfunction of cell cycle machinery and increased neurodegeneration in morphine using HIV infected patients as well as in the presence of HIV derived proteins (Krathwohl and Kaiser, 2004; Malik et al., 2014).

### Conflict of Interest Statement

The authors declare that the research was conducted in the absence of any commercial or financial relationships that could be construed as a potential conflict of interest.
